# Optimization of reaction temperature and Ni–W–Mo catalyst soaking time in oil upgrading: application to kinetic modeling of in-situ upgrading

**DOI:** 10.1038/s41598-023-31314-3

**Published:** 2023-04-15

**Authors:** Mahdi Abdi-Khanghah, Arezou Jafari, Goodarz Ahmadi, Abdolhossein Hemmati-Sarapardeh

**Affiliations:** 1 Chemical Engineering Department, Tarbiat Modares University, Tehran, Iran; 2 Department of Mechanical and Aeronautical Engineering, Clarkson University, Potsdam, NY 13699-5725 USA; 3 Department of Petroleum Engineering, Shahid Bahonar University of Kerman, Kerman, Iran; 4 State Key Laboratory of Petroleum Resources and Prospecting, China University of Petroleum (Beijing), Beijing, China

**Keywords:** Chemical engineering, Crude oil

## Abstract

Decreasing the conventional sources of oil reservoirs attracts researchers’ attention to the tertiary recovery of oil reservoirs, such as in-situ catalytic upgrading. In this contribution, the response surface methodology (RSM) approach and multi-objective optimization were utilized to investigate the effect of reaction temperature and catalysts soaking time on the concentration distribution of upgraded oil samples. To this end, 22 sets of experimental oil upgrading over Ni–W–Mo catalyst were utilized for the statistical modeling. Then, optimization based on the minimum reaction temperature, catalysts soaking time, gas, and residue wt.% was performed. Also, correlations for the prediction of concentration of different fractions (residue, vacuum gas oil (VGO), distillate, naphtha, and gases) as a function of independent factors were developed. Statistical results revealed that RSM model is in good agreement with experimental data and high coefficients of determination (R^2^ = 0.96, 0.945, 0.97, 0.996, 0.89) are the witness for this claim. Finally, based on multi-objective optimization, 378.81 °C and 17.31 h were obtained as the optimum upgrading condition. In this condition, the composition of residue, VGO, distillate, naphtha, and gases are 6.798%, 39.23%, 32.93%, 16.865%, and 2.896%, respectively, and the optimum condition is worthwhile for the pilot and industrial application of catalyst injection during in-situ oil upgrading.

## Introduction

The development of industries has been influenced by the availability of hydrocarbon sources such as crude oil, and the gradual increase of hydrocarbon requirement leads to the exploration of new techniques for oil production from oil reservoirs^1,2^. Due to limitation of the conventional oil reservoirs and natural or primary production restrictions, production from unconventional or heavy oil reservoirs is crucial^3–5^.

Thermal enhanced oil recovery, gas injection, in-situ combustion, and chemical injections are examples of the oil recovery methods for unconventional oil reservoirs^6^. The mentioned methods confront with feasibility, technical, and environmental limitations in experimental and pilot scale analyses^7,8^. However, in-situ catalytic upgrading has been introduced as one of the most economically beneficial and high-tech methods^9,10^. In this method, different nano and micro-sized materials are used as the catalyst source. These materials, which are named catalysts^11^, not only decrease oil viscosity followed by increasing the mobility ratio of oil in porous media, but also increase heavy oil quality. The quality of heavy oil depends on the amount of heavy fraction of oil (based on boiling point distribution such as residue and vacuum gas oil (VGO)) and light fractions (such as distillate and naphtha)^12,13^.

The mechanism of oil upgrading based on the conversion of heavy fractions to light fractions depends on experimental factors such as catalyst type^14^, upgrading reaction temperature and reaction time, and hydrogen donor agent in the reaction environment^15^. Based on different researches, Ni–W–Mo based catalysts were introduced as the best catalyst with the highest performance in oil upgrading reactions in the open literature^16,17^. Effect of nickel metal in the three metallic Ni–W–Mo was investigated by Wang et al.^18^. They reported that increasing nickel content, directly affects surface area, catalytic performance, catalytic activity, and pore size distribution of synthesized catalysts. Also, they found that increasing nickel content up to a specific criterion has positive effect on the upgrading reaction and providing excess nickel metals in the catalysts causes the deactivation of active upgrading sites on the catalysts^19^. In other words, it was found that increasing Ni content leads to attachment of Ni to other metal such as W and Mo as the sintering effect. To eliminate sintering effect, optimum catalyst formulation was investigated with experimental evaluations^20^. Among three types of metals in the selected catalysts, Ni, W and Mo play a vital role in hydro-cracking, hydrogenation and isomerization reaction, respectively^20^. Therefore, many researches have been published on finding optimum ratio of these three metals in the upgrading catalysts^21–23^. Optimum catalysts formulation for the three metallic Ni–W–Mo reported by Gallarag et al.^20^ was Ni/Me(atomic) = 0.3 with, Me = Ni + W + Mo, and Mo/W(atomic) = 3. Consequently, all data used in this research belongs to upgrading data over optimum formulation catalysts. On the other hand, reaction temperature and reaction time known as the catalysts soaking time in oil reservoirs, which strongly affect the economic feasibility of in-situ upgrading, was not optimized. Finding optimum reaction conditions followed by optimum composition assist reservoir simulation and economic analysis of in-situ upgrading. In order to find optimum condition, there are different multi objective optimization algorithms published in previous researches. Also, there are^24–26^ different kinetic studies evaluating composition variation with time during oil upgrading. Kinetic evaluations provide detail of composition variation with time^16,20,27–32^. However, in order to find the optimum composition, it is essential to model the composition variation with time using statistical approaches^21^. But there is not any predictive model for the estimation of composition distribution of upgraded samples as a function of operational parameters. In this research, optimization of reaction time and reaction temperature for oil upgrading over Ni–W–Mo catalysts is performed. It is clear that based on the utilized experimental data presence of hydrogen for the upgrading reactions is essential. Therefore, optimization developed in this research is based on static reaction conditions and the presence of hydrogen. To make this process feasible for fluid injection in porous media, different scenarios can be considered. In some researches, substation of tetralin and decalin with hydrogen is applicable^33–35^. In other words, one can use tetralin and decalin as hydrogen donor agents in the upgrading medium. On the other hand, some other research has been published in the open literature for using water as the hydrogen donor agent^29,33,36^. Aquathermolysis reactions occur in the presence of water and catalysts at porous media. Therefore, to make upgrading reaction and optimized condition similar to the feasible scenarios without hydrogen, it can be recommended to use different hydrogen donor agents such as tetralin, decalin, or water. Since other feasible conditions for reservoir condition mentioned above did not investigate composition variation, optimization of reaction time and reaction temperature was used for hydrogen included upgrading conditions.

In the present study, experimental oil upgrading data over Ni–W–Mo catalysts are used for the response surface methodology (RSM) modeling and optimization. In this regard, two independent factors (reaction temperature and catalysts soaking time) and five dependent targets (residue wt.%, VGO wt.%, distillate wt.%, naphtha wt.%, and gases wt.%) are considered for RSM modeling. 22 experimental data sets of different oil upgrading reactions over Ni–W–Mo catalysts are utilized and correlations based on coded factors and actual values of factors are developed. Moreover, multi-objective optimization based on statistical algorithms is performed. Finally, the effect of reaction temperature and catalysts soaking time on the concentration distribution of upgraded samples is investigated.

## Modeling procedure

### Response surface methodology approach

RSM is a set of numerical and analytical techniques for experimental design^37^. This method could prepare model development, parameter evaluation, and condition optimization. Also, the sensitivity analysis of the optimal conditions can be determined according to the various variables^38–40^. This method is applied for optimization to overcome expensive experimental costs. Another improvement is practicing graphical demonstrations and analyzing the relationships between variables and responses^41^. The RSM approach consists of full factorial or fractional factorial design, additional design, and a central point. By these modalities, information can be provided in the form of cubic or quadratic diagrams^42,43^. Standard statistical software would count the exact access of the model. Input levels of different variables for a specific level of response were specified in the RSM model. To discover a critical point (maximum, minimum, or saddle), it is essential for the polynomial function according to the latter equations should have cubic or quadratic terms^44^:

1 $$Quadratic\; model: Y={\beta }_{0}+\sum_{i=1}^{k}{\beta }_{i}{x}_{i}++\sum_{i=1}^{k}\sum_{j=1}^{k}{\beta }_{0}{x}_{i}{x}_{j}$$

2 $$Cubic\; model: Y={\beta }_{0}+\sum_{i=1}^{k}{\beta }_{i}{x}_{i}++\sum_{i=1}^{k}\sum_{j=1}^{k}{\beta }_{0}{x}_{i}{x}_{j}+\sum_{i=1}^{k}\sum_{j=1}^{k}\times \sum_{z=1}^{k}{\beta }_{ijz}{x}_{z}{x}_{i}{x}_{j}$$

In these models, Y is the predictor-dependent variable, i.e., the composition of reactant and products during heavy oil upgrading reactions such as residue, vacuum gas oil (VGO), naphtha, distillate, and gases. Moreover, X attains for independent variables, i.e., reaction temperature and reaction time related to upgrading reactions^45^.

### Optimization

**Figure 1:**
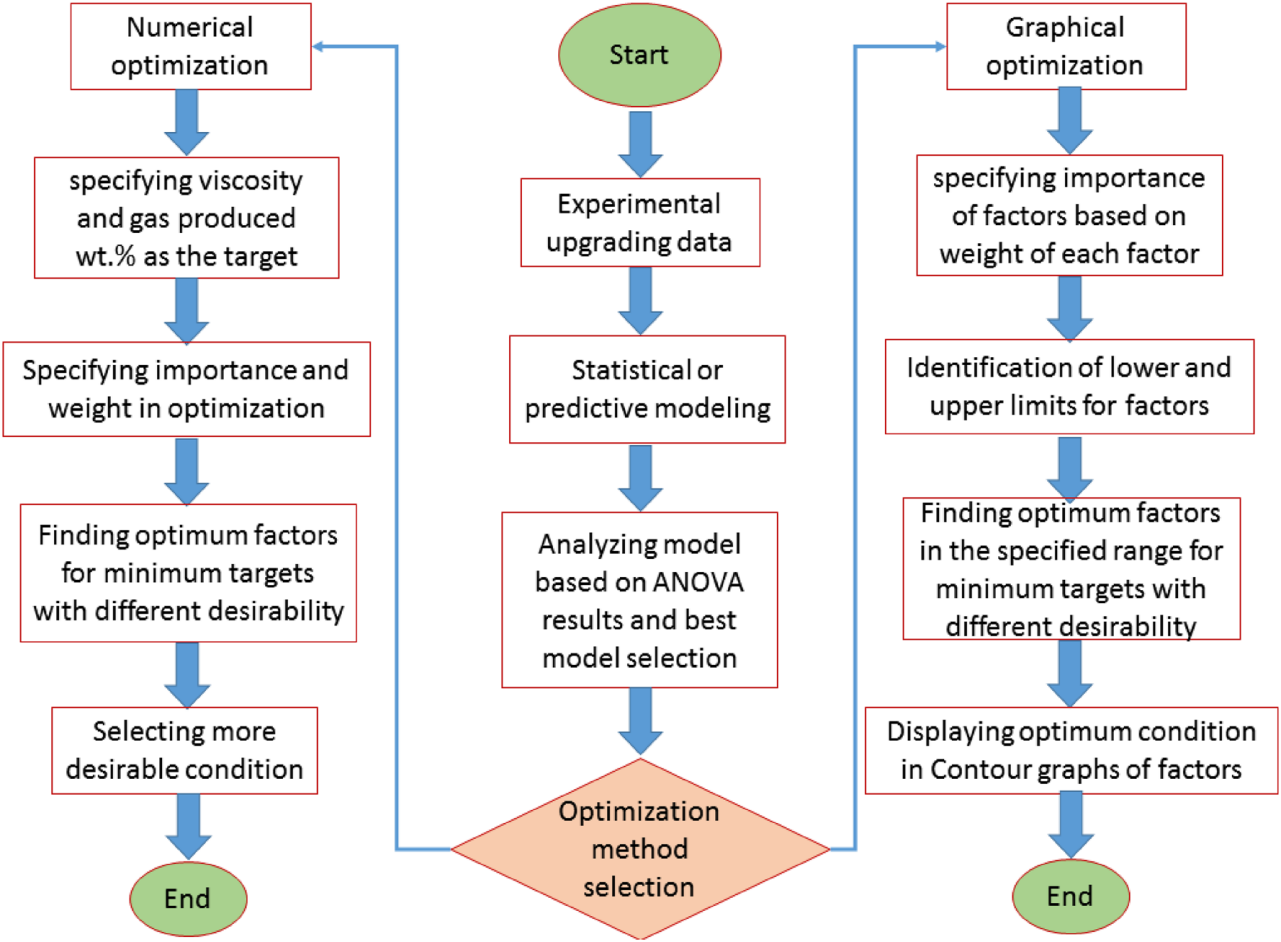
Schematic of optimization algorithm to find optimum catalysts soaking time and reaction temperature.

The goal of response level optimization is to attain the aspired position in the design area. It could be a maximum, a minimum, or a situation where the response is constant across various factors^46^. This research used a simultaneous optimization procedure to optimize multiple results. Surfaces produced through linear models would be applied to characterize the direction of the original design to adjust the desired requirements. However, if the test area is not transferable for material or instrumental purposes, the research should visually examine the best-operating conditions within the inquired examination conditions^42^. Therefore, optimum temperature and reaction time with respect to suitable upgraded oil composition is introduced for heavy oil upgrading reactions. The schematic of the optimization algorithm to find optimum catalyst soaking time and the reaction temperature is shown in Fig. 1.

### Experimental upgrading data

Experimental upgrading data^16,20,47^ are reported in Table 1. To implement a statistical-based RSM model for predicting fraction concentration of upgraded oil as a function of temperature and time, 22 sets of data points were selected. All fractions were measured for the upgraded oil samples with Ni–W–Mo catalyst. The minimum, maximum, and average reaction temperature and catalysts soaking time used as the experimental data are 320, 400, 363.6 and 0, 69, 15.075, respectively. Also, crude oil used for the upgrading has the residue, VGO, distillate, naphtha, and gases wt.% of 47.8%, 34.5%, 15%, 2.8%, and 0%, respectively. Reaction temperature steps are 320, 350, 380, and 400 °C, while catalysts soaking time is distributed from 0 to 69 h. The utilized data sets were gathered from the catalytic oil upgrading investigation reported by Galaraga et al., Sanchez et al., and Ancheyta et al.^16,20,47^. Furthermore, the data set was used based on run order and random distribution in design expert software as the historical data category. Also, important notice is that composition of crude oil before upgrading reaction directly affects upgraded oil composition. Therefore, heavy oil composition (Residue, VGO, Distillate, Naphtha) for data used form the report of Sanchez et al. and Ancheyta et al.^15,28^ were considered as (47.8, 34.5, 15, 2.8). Also (24.64, 30.5, 22.42, 22.42) were assumed for the crude oil composition (Residue, VGO, Distillate, Naphtha) of data collected from the work reported by Galaraga et al.^12^.

**Table 1:** Experimental data for the in-situ upgrading products at different temperature and soaking time.

Run number	Oil type	Temperature (c)	Time (hr)	VGO wt.%	Distillate wt.%	Naphtha wt. %	Gas wt.%	Residue wt.%	Refs.
1	S1	320	0	34.5	15	2.8	0	47.8	^20^
2	S1	320	24	35.8	15.6	2.7	0.6	45.1	^20^
3	S1	320	38	35.8	16.1	3	0.4	44.4	^20^
4	S1	320	48	35.8	17	4.2	0.3	42.6	^20^
5	S1	320	69	37.7	17.7	3.3	0.1	41	^20^
6	S1	350	0	34.5	15	2.8	0	47.8	^20^
7	S1	350	6	35.3	17	4.6	0.1	42.9	^20^
8	S1	350	7	36.1	17.1	3.7	0.3	42.6	^20^
9	S1	350	22	38.9	20.3	4.7	0.8	35	^20^
10	S1	350	30	39.6	21.8	5.8	1.8	30.7	^20^
11	S1	350	48	38.7	22.7	7.9	2.2	28.1	^47^
12	S1	380	0	34.5	15	2.8	0	47.8	^47^
13	S1	380	3	38	20.3	6.7	2.2	32.6	^47^
14	S1	380	5.8	39.1	23.9	8.8	2	25.9	^47^
15	S1	380	8	40.2	27.1	10.9	2	19.5	^47^
16	S1	380	14	36.4	29.4	14.6	2	16.9	^47^
17	S2	400	0.6593	30.5	22.4286	22.4286	1.79904	22.8437	^16^
18	S2	400	0.7960	30.78	23.0714	23.0714	2.14593	20.9255	^16^
19	S2	400	1	32.14	24.1429	24.1429	2.56699	17.0043	^16^
20	S2	400	1.3244	32.21	25.5714	25.5714	3.33254	13.3103	^16^
21	S2	400	2	33.07	25.8571	25.8571	4.01435	11.2000	^16^
22	S2	400	3	33.85	26.8571	26.8571	4.86842	7.56028	^16^

## Results and discussion

Twenty-two experimental heavy oil upgrading data sets were utilized for RSM modeling. The statistical characteristics of these data were reported in Section "Experimental upgrading data" and all experimental data are provided in Table 1. As mentioned before, reaction temperature and nano-catalyst soaking time are the factors affecting the performance of in-situ upgrading. On the other hand, product distribution or weight percent of products is a demonstrator for the performance of in-situ upgrading. It is obvious that viscosity reduction and high-quality products are two main targets of in-situ upgrading, and both of them depend on the economic benefits of oil recovery. Although using an active catalyst that produces a large amount of C_1_–C_3_ reduces the viscosity of heavy oil, these products are not suitable from an economic aspect. In other words, producing middle products such as naphtha, distillate, and vacuum gas oil is more interesting than light gases from an economic aspect. Hence, there is an optimization problem. Maximum conversion of heavy oil and maximum production of naphtha, distillate, and vacuum gas oil, along with the minimum production of gases, is the optimization target. Hence, the effect of two factors, namely temperature and soaking time, on the weight percent of each product (responses) were considered. Then, various techniques for finding the correlation between factors, and responses were applied.

### Predictive models

In this step, quadratic and cubic correlations were used and their performance in correlating factors and responses were studied. Correlations between factors and responses based on actual and coded values are reported in Table 2. Moreover, actual and coded values of factors are reported in Table 3. Hence, one can calculate the amount of each fraction in different reaction temperatures and catalyst soaking times using actual data and coded correlations. Also, tables of analysis of variance (ANOVA) for each model and response were provided in the Supporting data file are provided in Tables S1–S5 of supporting data file. Moreover, in order to investigate the performance of developed statistical methods, some quantitative and qualitative methods were employed.

**Table 2:** Cubic and quadratic correlations between factors and weight percent of in-situ upgrading products based on coded and actual values.

Responses	Correlation based on coded values	Correlation based on actual values
VGO	$$\begin{aligned} VGO = & 39.74135418 - 0.97401393*A \\ & - \;3.362057513*B - 12.12089246*A*B \\ & - \;10.73174033*A^{2} - 6.321848098*B^{2} \\ & - \;8.881773282*A^{2} *B - 6.755433001*A*B^{2} \\ & - \;5.733901501*A^{3} 1.749554005*B^{3} \\ \end{aligned}$$	$$\begin{aligned} VGO = & 4025.675863 - 33.89118031*T \\ & - \;20.79429267*t + 0.116856444*T*t \\ & + \;0.095603358*T^{2} + 0.041359703*t^{2} \\ & - \;0.000160902*T^{2} *t - 0.000141891*T*t^{2} \\ & - \;8.95922E - 05*T^{3} + 4.26059E - 05*t^{3} \\ \end{aligned}$$
Distillate	$$\begin{aligned} Distillate = & 27.84715715 + 26.06072245*A \\ & + \;5.890007576*B + 17.2618268*A*B \\ & + \;16.55280908*A^{2} - 7.264024871*B^{2} \\ & + \;12.9762842*A^{2} *B - 7.654490192*A*B^{2} \\ & + \;2.294516374*A^{3} - 0.20849871*B^{3} \\ \end{aligned}$$	$$\begin{aligned} Distillate = & - \;1378.401948 + 12.35836092*T \\ & + \;22.54302368*t - 0.145653847*T*t \\ & - \;0.036484636*T^{2} + 0.052301519*t^{2} \\ & + \;0.000235078*T^{2} *t - 0.000160775*T*t^{2} \\ & + \;3.58518E - 05*T^{3} - 5.07746E - 06*t^{3} \\ \end{aligned}$$
Naphtha	$$\begin{aligned} Naphtha = & 10.78929741 + 24.10431878*A \\ & + \;9.308936562*B + 28.03149068*A*B \\ & + \;31.14461679*A^{2} - 1.08574458*B^{2} \\ & + \;20.28033669*A^{2} *B - 1.018261168*A*B^{2} \\ & + \;14.64984421*A^{3} - 1.220843124*B^{3} \\ \end{aligned}$$	$$\begin{aligned} Naphtha = & - 9753.60538 + 83.98524167*T \\ & + \;39.99747974*t - 0.242737716*T*t \\ & - \;0.240425946*T^{2} + 0.009864428*t^{2} \\ & + \;0.000367397*T^{2} *t - 2.13875E \\ & - \;05*T*t^{2} + 0.000228904*T^{3} \\ & - \;2.97305E - 05*t^{3} \\ \end{aligned}$$
Gas	$$\begin{aligned} Gas = & 2.57941127 + 3.699912999*A \\ & + \;2.25930855*B + 2.352934734*A*B \\ & + \;1.612955841*A^{2} - 0.418884258*B^{2} \\ \end{aligned}$$	$$\begin{aligned} Gas = & 118.4278372 - 0.692155672*T \\ & - \;0.524038711*t0.001705025*T*t \\ & + \;0.001008097*T^{2} - 0.00035193*t^{2} \\ \end{aligned}$$
Residue	$$\begin{aligned} Residue = & 18.31280692 - 55.97139703*A \\ & - \;14.9020041*B - 40.57833961*A*B \\ & - \;42.12921684*A^{2} + 14.31108797*B^{2} \\ & - \;28.21339801*A^{2} *B + 14.46116375*A*B^{2} \\ & - \;12.1951362*A^{3} - 1.038863196*B^{3} \\ \end{aligned}$$	$$\begin{aligned} Residue = & 7820.02481 - 67.84663161*T \\ & - \;49.4614494*t + 0.317638041*T*t \\ & + \;0.197095538*T^{2} - 0.094705143*t^{2} \\ & - \;0.00051111*T^{2} *t + 0.000303742*T*t^{2} \\ & - \;0.000190549*T^{3} - 2.52989E - 05*t^{3} \\ \end{aligned}$$

**Table 3:** Statistical analysis of the factors and coded values.

Factor	Name	Units	Type	Low actual	High actual	Low coded	High coded	Mean	Std. Dev
A	Temp	(°C)	Numeric	320	400	− 1	1	363.6364	30.08253
B	Time	hr	Numeric	0	69	− 1	1	15.07181	19.28253

**Figure 2:**
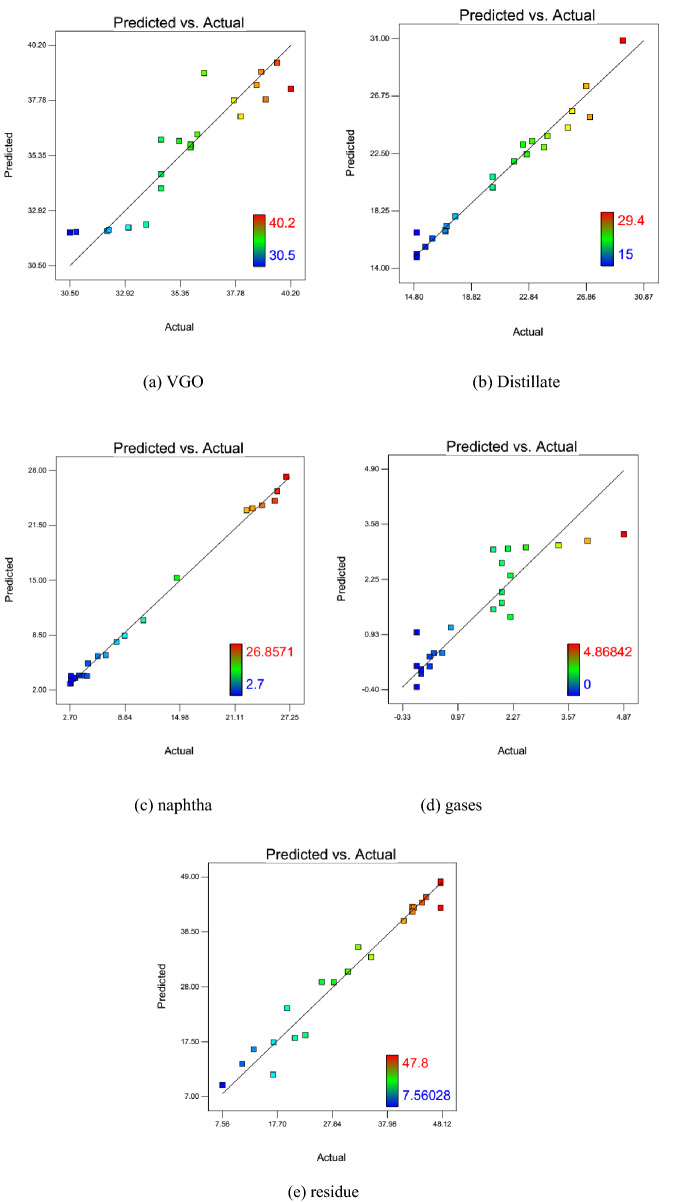
Predicted versus actual values of weight percent of in-situ upgrading products (**a**) VGO, (**b**) Distillate (**c**) Naphtha, (**d**) Gasses, and (**e**) residue.

Figure 2a–e illustrate the predicted versus actual values of VGO, distillate, naphtha, gases, and residue, respectively. The scatter of data points in the mentioned figures around the Y = X or diagonal line are a great witness for agreement between experimental and predicted values by the statistical models. Therefore, one can conclude that the developed RSM model can greatly predict the weight percent of oil fractions during oil upgrading.

#### VGO

**Figure 3:**
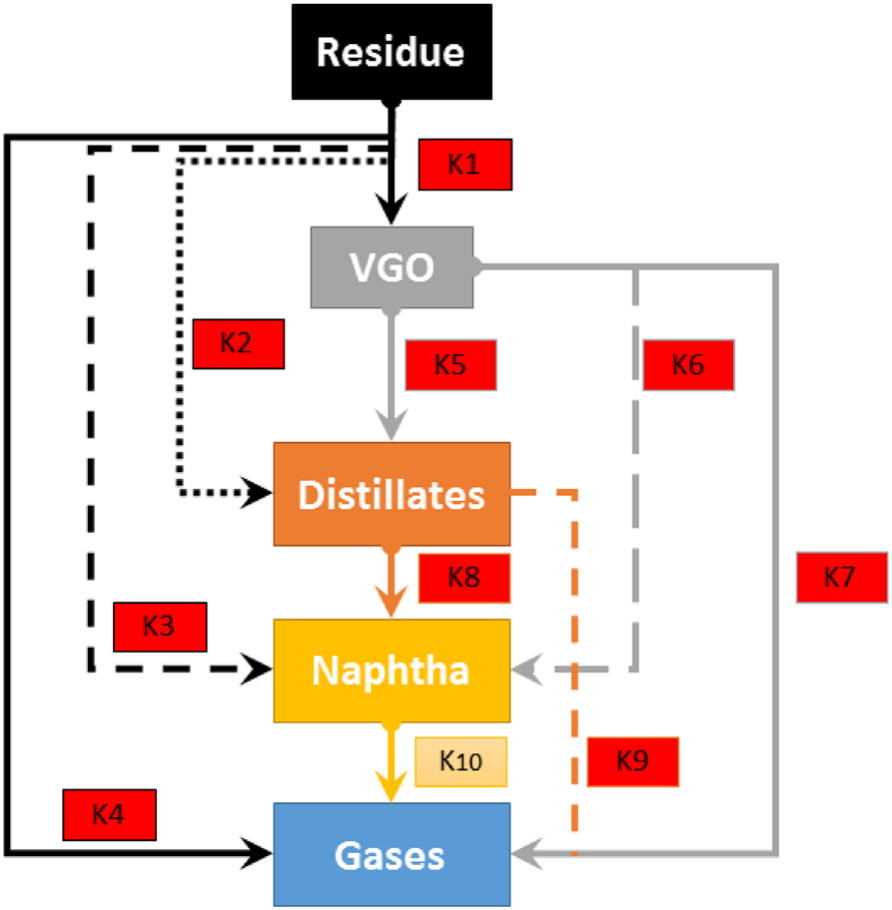
Reaction network for the oil upgrading based on 5 lumped components including 10 irreversible reactions^21^.

The following equation demonstrates the correlation between factors and VGO w.t% during oil upgrading.

3 $${\text{VGO wt}}\% = { 39}.{74135418} - 0.{974}0{1393}*{\text{A}} - {3}.{362}0{57513}*{\text{ B}} - {12}.{12}0{89246}*{\text{ A}}*{\text{B}}$$

where A and B denote the reaction temperature and catalysts soaking time, respectively. The mentioned correlation is based on coded values of factors reported in Table 2. As can be understood from the correlation, the sign of coefficients for both the reaction temperature and catalysts soaking time is negative. Therefore, the higher the reaction temperature and catalysts soaking time, the lower the values of VGO wt.% in upgraded oil. The reaction network for the oil upgrading based on five lumped components is shown in Fig. 3. As can be understood from the reaction network, VGO is one of the heavy fractions of oil which is exposed to upgrading during catalytic reactions. Therefore, it is clear that increasing catalysts soaking time leads to progress of upgrading reaction followed by decreasing VGO wt.%. Moreover, increasing reaction temperature results in the increment of the reaction rate constants of VGO conversion toward light fraction production based on the Arrhenius equation. For more elaboration, Arrhenius equation is expressed as follows:

4 $$K = K_{0} *{\text{exp}}\left( {\frac{{ - E}}{{RT}}} \right)$$

where K_0_, E, R, and T are the pre-exponential factors, reaction rate activation energy, the universal gas constant, and reaction temperature. It is evident that increasing reaction temperature increases exponential term followed by increasing reaction rate constant. For the VGO fraction, there is a production and three conversion reactions. Therefore, the negative sign of coefficient in the correlation developed for VGO illustrates that at the elevated temperature of upgrading reaction, conversion rates are higher than the production rate from residue.

The negative sign of the interaction parameter (interaction between reaction temperature and catalyst soaking time) proves that not only each factor is in the opposite direction of VGO wt.%, but also the interaction of them causes the reduction. In other words, both the reaction temperature and catalysts soaking time expedite the single effect of factors in decreasing VGO concentration during oil upgrading. Another reliable understanding from the developed correlation is that the coefficient of factor B is more than Factor A. This comparison indicates that catalyst soaking time is more effective than reaction temperature in reducing VGO wt.% during oil upgrading.

#### Distillate

Correlations for the prediction of distillate wt.% as a function of factors based on actual and coded values are provided in Eqs. (5) and (6), respectively.

5 $$\mathrm{Distillate\; wt\% }= -1378.401948+ 12.35836092*\mathrm{ Temp}+22.54302368*\mathrm{time}-0.145653847*\mathrm{Temp}*\mathrm{time}$$

6 $$Distillate\; wt\%= 27.84715715+ 26.06072245*A+ 5.890007576* B+ 17.2618268*A*B$$

where temp, time, A, and B are the actual values of reaction temperature, actual values of catalysts soaking time, coded factor of reaction temperature, and coded factor of catalyst soaking time, respectively. Between these correlations, an equation based on coded factors provides detailed information about the factors. The coefficients of both factor A and B have a positive sign in the correlation based on coded values. Therefore, the higher the reaction temperature, the more the distillate production. In other words, at a higher reaction temperature speed of distillate production from residue and VGO are more than its conversion to naphtha and gases. The mentioned phenomena can be described with the Arrhenius equation provided in Eq. (4). The trends observed for the effect of reaction temperature and catalyst soaking time on the distillate and VGO concentration are against each other. Increasing A and B increases distillate wt.%; however, VGO wt.% decreases with increasing reaction temperature and catalyst soaking time. Therefore, one can conclude that increasing effective factors increases conversion and production of VGO and distillate, respectively.

Coefficients of A and B in the coded correlation for the prediction distillate are 26.06 and 5.89, respectively. Values of coefficients illustrate that reaction temperature has more influence on distillate wt.% than catalysts soaking time. However, in the VGO wt.%, catalyst soaking time is more influential than reaction temperature. Consequently, not only effects of factors A and B on distillate wt.% and VGO wt.% (based on negative and positive signs in coded correlation) are dissimilar, but also the more effective factors on the mentioned concentrations in upgraded oil are different.

#### Naphtha and gases fractions

The RSM model fitted correlations on the responses of naphtha wt.% and gas wt.% as a function of independent factors as follows:

7 $$\mathrm{Naphtha\; wt\%}=10.78929741+24.104*\mathrm{A}+ 9.308936562*\mathrm{ B}+28.03*\mathrm{A}*\mathrm{B}$$

8 $$\mathrm{Gas}= 2.57941127 +3.699912999*\mathrm{A}+ 2.259*\mathrm{ B}+ 2.352934734*\mathrm{A}*\mathrm{ B}$$

Equations (7) and (8) are the coded correlation for the naphtha wt.% and gases wt.%, respectively. Since the coefficients of A and B in both equations are positive, the effect of factors A and B on the naphtha wt.% and gases wt.% are similar to the distillate wt.%. Therefore, distillate, naphtha, and gases can be considered as the product of the reaction network presented in Fig. 3. Furthermore, the coefficients of factor A (reaction temperature) in both Eqs. (7) and (8) (24.104 and 3.69) are more than that of factor B (catalysts soaking time) in both Eqs. (7) and (8) (9.308 and 2.25). Consequently, reaction temperature shows a higher impact on the concentration of naphtha and gases than the catalysts soaking time. The mentioned trend is similar to the observed trend for the distillate wt.%. Hence, one can conclude that for the three fractions whose production rate is more than the consumption rate, the reaction temperature shows a superior influence on their concentration increment.

Also, the coefficients of interaction (A*B) in both equations are positive. Similarly, positive values were calculated for fitting distillate wt.% by the RSM model. Hence, the interaction between catalysts soaking time and reaction temperature causes the increase of their impact on the production rate of three fractions named products. In other words, reaction temperature supports the impact of catalyst soaking time to increase the production rate of distillate, naphtha, and, gases, and similarly, catalyst soaking time pushes reaction temperature to this end.

#### Residue

The following correlations were derived for the prediction of residue with actual and coded factors.

9 $$\mathrm{Residue}= 18.31280692-55.971*\mathrm{A }-14.90*\mathrm{B }-40.578*\mathrm{A}*\mathrm{B}$$

10 $$\mathrm{Residue}= 7820.0248-67.8466*\mathrm{Temp }-49.461*\mathrm{ time}+ 0.3176*\mathrm{ Temp}*\mathrm{ time}$$

Coded and actual correlations are reported in Eqs. (9) and (10), respectively. Substituting actual values of reaction temperature (°C) and catalyst soaking time (hr) in Eq. (2) leads to the calculation of residue based on actual factors. However, this correlation and its coefficients do not illustrate any physical meaning. Therefore, coded correlation assists in comprehending any physical phenomena during oil upgrading reactions. As can be seen in Eq. (9), the coefficients of factor A, B, and AB has a negative sign. Therefore, increasing reaction temperature, catalysts soaking time, and their interaction result in the decrease of residue wt.%. As shown in reaction network in Fig. 3, residue is considered as a fraction with no production during upgrading reactions. Residue fraction consumes or upgrades during upgrading reaction to other four fractions named VGO, distillate, naphtha, gases. Increasing reaction temperature increases the exponential term of the Arrhenius equation followed by increasing reaction rate constants for the consumption of residue. Therefore, a negative sign for the reaction temperature is in good agreement with the reaction network and upgrading reaction mechanism. On the other side, the higher the reaction time, the more the reaction conversion followed by residue wt.% reduction. This phenomenon confirms a negative sign in the coded correlation of residue wt.%. As the final consequence of the coded correlation, it is evident that the interaction between factor A and factor B has a negative sign, which shows the aligned effect of reaction temperature and catalysts soaking time on residue wt.%.

### Contour plots

**Figure 4:**
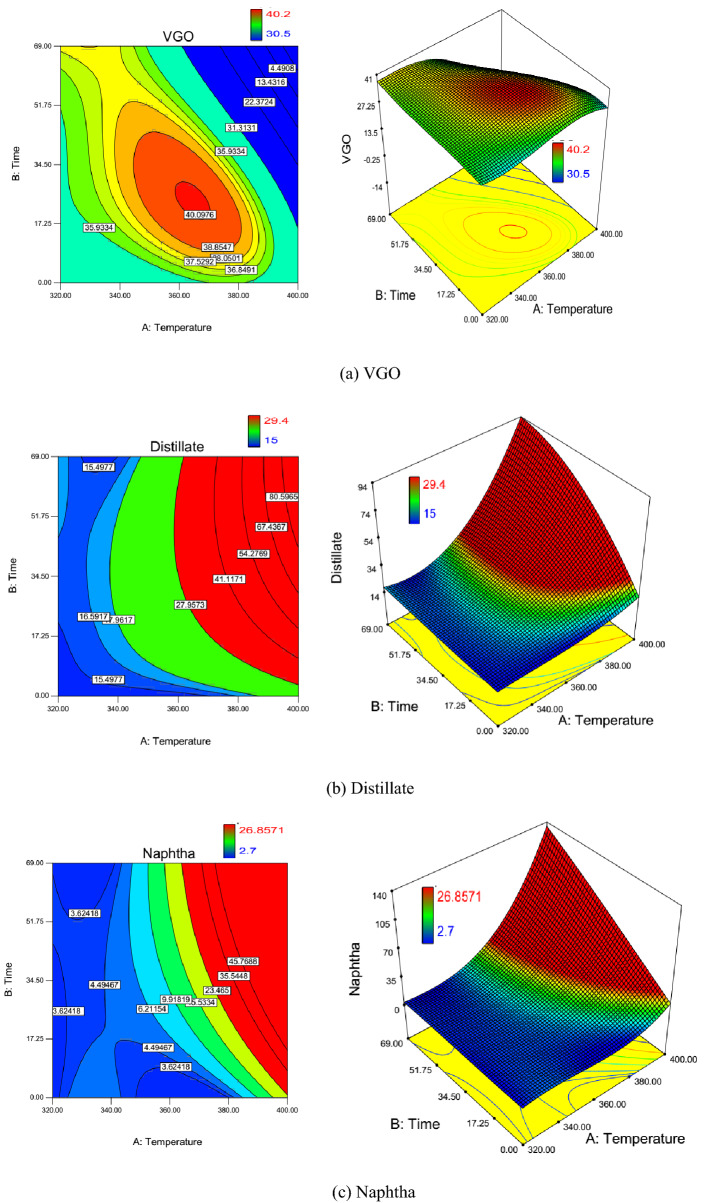

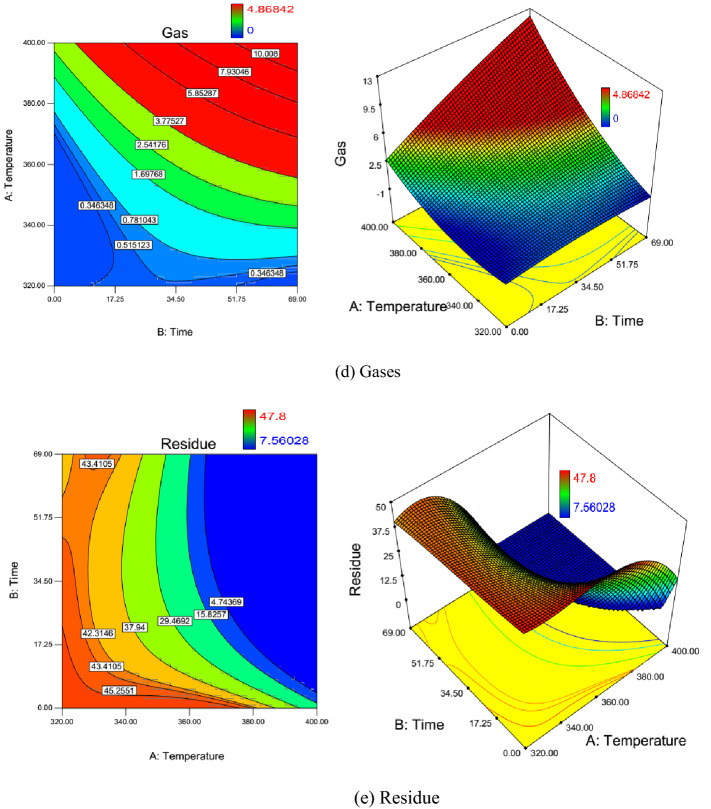
2D and 3D contour plots of the effect of temperature and catalyst soaking time on the weight percent of in-situ upgrading products (**a**) VGO, (**b**) Distillate (**c**) Naphtha, (**d**) Gases, and (**e**) Residue.

Two-dimensional contour plots and three-dimensional surface view for the effect of reaction temperature and catalysts soaking time on the VGO wt.%, distillate wt.%, naphtha wt.%, gases wt.%, and residue wt.% during oil upgrading with catalysts are presented in Fig. 4a–e. Color bar indication beside the two-dimensional contour plot of VGO wt.% shown in Fig. 4a, illustrates that minimum and maximum weight percent of VGO during upgrading reaction are 30.5% (blue) and 40.2% (red), respectively. Color variation from red to blue illuminates VGO concentration reduction. As shown in this figure, from the left bottom corner of the contour plot (zero reaction time) to the right side (increasing reaction time), VGO concentration increases and then decreases gradually. This mechanism occurs due to the dominance of the rate of residue conversion to VGO and VGO conversion to the lighter fraction in the increase and decrease section of VGO wt.%, respectively. Hence, one can conclude that up to the reaction temperature of 370 °C dominant reaction mechanism is the conversion of residue to VGO.

Figure 4b demonstrates the effect of reaction temperature and catalysts soaking time on distillate wt.%. 29.4% and 15% are the maximum and minimum values of distillate wt.% which can be observed in the maximum reaction condition (maximum reaction temperature and catalysts soaking time) and crude oil concentration. Therefore, with a simultaneous increase of reaction temperature and catalysts soaking time distillate concentration increases. The mentioned trend illustrates that interaction between two factors is in the same direction as the distillate production. Also, interaction coefficient term in the coded correlation developed for the prediction of distillate was positive. Accordingly, the trend observed in the two-dimensional contour plot are in agreement with the coefficient of the interaction of factors in the coded correlation. Another important note is that in the constant reaction temperature, increasing catalysts soaking time leads to distillate concentration increment up to 34 h. However, after 34 h. and up to 69 h. of catalysts soaking time, increasing catalyst soaking time results in the reduction of distillate concentration. Therefore, at the constant reaction temperature, the dominant reaction mechanism before 34 h. catalyst soaking time is the production of distillate from residue and VGO (see reaction network in Fig. 3) and after the mentioned catalyst soaking time conversion of distillate to naphtha and gases overcome the speed of distillate production.

Naphtha and gases are the lightest fractions of hydrocarbons in which their variation during upgrading reactions is shown in Fig. 4c,d, respectively. Minimum and maximum of naphtha and gases based on color variation are (2.7–26.8) and (0–4.86), respectively. Since crude oil does not contain any light hydrocarbon (C_1_–C_3_) in the atmospheric condition, the minimum values of gas are zero which is measured for the crude oil composition. Variation trends for both gases and naphtha are similar to each other. From the left bottom corner (minimum reaction temperature and catalysts soaking time) to the right top corner (maximum reaction temperature and catalysts soaking time) of the contour plot, the blue color changes to red. This color variation shows that the higher the reaction temperature and catalysts soaking time, the higher the concentration of naphtha and gases. The mentioned mechanism can be described using the upgrading reaction network shown in Fig. 3. Naphtha and gases are at the end of the reaction path in the reaction network. Since the distribution of concentration of naphtha and gases are similar, the difference between naphtha production from heavier fractions and naphtha conversion to gases (net naphtha wt.% increase) is similar to the rate of production of gases from all heavier fractions (net gases wt.% increase).

### Optimization procedure

The main objective of RSM modeling and optimization of upgrading reactions was to achieve minimum gas production in minimum reaction temperature and catalysts soaking time along with maximum residue conversion. Multi-objective optimization has been applied to find optimal reaction conditions. Range of factors and targets and criteria implemented in the optimization is provided in Table 4. As listed in Table 4, minimization of concentration of residue (progress of upgrading reactions), gases (avoid the production of hydrocarbon with low economic value), catalysts soaking time (lower operational time for upgrading), and reaction temperature (lower operational cost) are the major assumption for the multi-objective optimization in this research. The importance of all variables was set at 3 except residue wt.% which was set at 5 grade importance. This adjustment on the residue shows that residue conversion is more important than the other optimization targets. Optimum factors and corresponding targets at optimum reaction temperature and catalysts soaking time are reported in Table 5. Two optimum conditions were selected based on their desirability. First row of Table 5 reveals that at reaction temperature and catalyst soaking time of 378.81 °C and 17.31 h, optimum condition of different fractions occurs during oil upgrading with catalyst. Residue wt.% reduced to 6.79% during this condition which is a very interesting result for reducing heavy components of oil; however, 2.89% of upgraded oil converts to gases in the optimum condition reported in the first of Table 5. In addition, the second row of the mentioned table illustrates that 40.828% and 0.012% of residue and gas wt.% can be achievable at upgrading temperature of 320 °C and 67.7 h. The second choice is more interesting from the gas wt.% reduction. However, the amount of residue did not reduce enough during upgrading temperature of 320 °C and catalysts soaking time of 67.7 h. Consequently, between the two choices which have the highest desirability (near the unity), the first row (378.81 °C and 17.31 h) is introduced as the optimum upgrading condition. In this condition, composition of residue, VGO, distillate, naphtha, and gases are 6.798%, 39.23%, 32.93%, 16.865%, and 2.896%, respectively. It should be mentioned coke formulation over catalysts during in-situ upgrading is undeniable. As can be seen in the composition data with time and temperature, a sharp reduction of residue (major reactant in heavy oil composition) can be observed at the early times of reaction. However, residue concentration reduction (composition difference/time interval) reduces with increasing time. This shows that catalysts activity decreases over time and one of the major reasons of this phenomena is the coke laid down over the catalysts surface.

**Table 4:** Criteria used for the optimization of upgrading reaction condition.

Response	Name	Minimum	Maximum	Mean	Objective	Importance
Y1	VGO	30.5	40.2	35.61234	In range	–
Y2	Distillate	15	29.4	20.86039	In range	–
Y3	Naphtha	2.7	26.8571	10.78311	In range	–
Y4	Gas	0	4.86842	1.523967	Minimum	3
Y5	Residue	7.56028	47.8	31.0702	Minimum	5
X1	Temperature	320	400	363.6	Minimum	3
X2	Time	0	69	15.07	Minimum	3

**Table 5:** Optimum values of factors and corresponding responses.

#	Temperature	Time	VGO	Distillate	Naphtha	Gas	Residue	Desirability
1	378.81	17.3	39.23	32.93	16.865	2.896	6.798	0.99
2	320	67.7	37.57	17.76	3.523	0.012	40.828	0.98

## Conclusions

In this contribution, experimental oil upgrading data sets over catalysts were used for the RSM modeling and optimization. In this regard, two independent factors (reaction temperature and catalysts soaking time) and five dependent targets (residue wt%, VGO wt.%, distillate wt.%, naphtha wt.%, and gases wt.%) were considered for RSM modeling. The significant outcomes of this research can be expressed as follows:

- The negative sign of coefficient in the correlation developed for VGO illustrates that at the elevated temperature of upgrading reaction, conversion rates are higher than the production rate from residue.
- Not only effects of factors A (reaction temperature) and B (catalysts soaking time) on distillate wt.% and VGO wt.% (based on negative and positive signs in coded correlation) are dissimilar, but also the more effective factors on the mentioned concentrations in upgraded oil are different.
- Naphtha and gases were located at the end of the reaction path in the reaction network. Since the distribution of concentration of naphtha and gases are similar, the difference between naphtha production from heavier fractions and naphtha conversion to gases (net naphtha wt.% increase) is similar to the rate of production of gases from all heavier fractions (net gases wt.% increase).
- Based on multi-objective optimization, 378.81 °C and 17.31 h are introduced as the optimum upgrading condition. In this condition, the composition of residue, VGO, distillate, naphtha, and gases are 6.798%, 39.23%, 32.93%, 16.865%, and 2.896%, respectively.

The mentioned conclusions illustrate the feasibility of this research for in-situ upgrading, However, catalyst injectivity in porous media is still challenging issue. The main recommendation of this research for future researches is to utilize different synthesis procedures such as micro emulsion synthesis to make catalysts injection feasible in porous media.

## Supplementary Information

Supplementary Tables.

## Data Availability

The datasets used and/or analyzed during the current study available from the corresponding author on reasonable request.
