# Supplementary material for: Optimization of reaction temperature and Ni–W–Mo catalyst soaking time in oil upgrading: application to kinetic modeling of in-situ upgrading
Source: Sci Rep. 2023 Apr 15;13:6158. doi: 10.1038/s41598-023-31314-3 (PMC10105742; doi:10.1038/s41598-023-31314-3)
Supplement: Supplementary file 1 — Supplementary Tables. [file 41598_2023_31314_MOESM1_ESM.docx]

Table S1. ANOVA table for the response VGO weight percent

| Analysis of variance table [Partial sum of squares - Type III] | | | | | |
| --- | --- | --- | --- | --- | --- |
| Source | Sum of  Squares | df | Mean  Square | F  Value | p-value |
| Model | 143.8098 | 9 | 15.97887 | 8.325814 | 0.0006 |
| A-Temperature | 0.060298 | 1 | 0.060298 | 0.031418 | 0.8623 |
| B-Time | 1.370744 | 1 | 1.370744 | 0.714228 | 0.4146 |
| AB | 2.3998 | 1 | 2.3998 | 1.250419 | 0.2854 |
| A^2 | 5.879325 | 1 | 5.879325 | 3.063431 | 0.1056 |
| B^2 | 2.60102 | 1 | 2.60102 | 1.355266 | 0.2670 |
| A^2B | 3.35676 | 1 | 3.35676 | 1.749045 | 0.2106 |
| AB^2 | 2.613241 | 1 | 2.613241 | 1.361633 | 0.2659 |
| A^3 | 14.55252 | 1 | 14.55252 | 7.582615 | 0.0175 |
| B^3 | 0.578498 | 1 | 0.578498 | 0.301427 | 0.5931 |
| Residual | 23.03035 | 12 | 1.919196 |  |  |
| Cor Total | 166.8402 | 21 |  |  |  |

Table S2. ANOVA table for the response Distillate weight percent

| Analysis of variance table [Partial sum of squares - Type III] | | | | | |
| --- | --- | --- | --- | --- | --- |
| Source | Sum of  Squares | df | Mean  Square | F  Value | p-value |
| Model | 422.0124 | 9 | 46.89027 | 44.9324 | < 0.0001 |
| A-Temperature | 43.16644 | 1 | 43.16644 | 41.36406 | < 0.0001 |
| B-Time | 4.207052 | 1 | 4.207052 | 4.03139 | 0.0677 |
| AB | 4.867201 | 1 | 4.867201 | 4.663975 | 0.0517 |
| A^2 | 13.98719 | 1 | 13.98719 | 13.40317 | 0.0033 |
| B^2 | 3.434079 | 1 | 3.434079 | 3.290691 | 0.0947 |
| A^2B | 7.165089 | 1 | 7.165089 | 6.865916 | 0.0224 |
| AB^2 | 3.355101 | 1 | 3.355101 | 3.215012 | 0.0982 |
| A^3 | 2.330345 | 1 | 2.330345 | 2.233043 | 0.1609 |
| B^3 | 0.008216 | 1 | 0.008216 | 0.007873 | 0.9308 |
| Residual | 12.52288 | 12 | 1.043574 |  |  |
| Cor Total | 434.5353 | 21 |  |  |  |

Table S3. ANOVA table for the response Naphta weight percent

| Analysis of variance table [Partial sum of squares - Type III] | | | | | |
| --- | --- | --- | --- | --- | --- |
| Source | Sum of  Squares | df | Mean  Square | F- Value | p-value |
| Model | 1773.036 | 9 | 197.004 | 361.8486 | < 0.0001 |
| A-Temperature | 36.92862 | 1 | 36.92862 | 67.82892 | < 0.0001 |
| B-Time | 10.50864 | 1 | 10.50864 | 19.30182 | 0.0009 |
| AB | 12.83507 | 1 | 12.83507 | 23.57491 | 0.0004 |
| A^2 | 49.51687 | 1 | 49.51687 | 90.95048 | < 0.0001 |
| B^2 | 0.07672 | 1 | 0.07672 | 0.140917 | 0.7139 |
| A^2B | 17.50134 | 1 | 17.50134 | 32.14571 | 0.0001 |
| AB^2 | 0.059373 | 1 | 0.059373 | 0.109054 | 0.7469 |
| A^3 | 94.99569 | 1 | 94.99569 | 174.484 | < 0.0001 |
| B^3 | 0.281687 | 1 | 0.281687 | 0.51739 | 0.4857 |
| Residual | 6.533252 | 12 | 0.544438 |  |  |
| Cor Total | 1779.569 | 21 |  |  |  |

Table S4. ANOVA table for the response Gases weight percent

| Analysis of variance table [Partial sum of squares - Type III] | | | | | |
| --- | --- | --- | --- | --- | --- |
| Source | Sum of  Squares | df | Mean  Square | F  Value | p-value |
| Model | 32.54954 | 5 | 6.509907 | 13.03716 | < 0.0001 |
| A-Temperature | 9.380343 | 1 | 9.380343 | 18.78568 | 0.0005 |
| B-Time | 3.206164 | 1 | 3.206164 | 6.420871 | 0.0221 |
| AB | 2.605865 | 1 | 2.605865 | 5.218673 | 0.0363 |
| A^2 | 5.058007 | 1 | 5.058007 | 10.12949 | 0.0058 |
| B^2 | 0.218725 | 1 | 0.218725 | 0.438032 | 0.5175 |
| Residual | 7.989356 | 16 | 0.499335 |  |  |
| Cor Total | 40.53889 | 21 |  |  |  |

Table S5. ANOVA table for the response Residue weight percent

| Analysis of variance table [Partial sum of squares - Type III] | | | | | |
| --- | --- | --- | --- | --- | --- |
| Source | Sum of  Squares | df | Mean  Square | F-Value | p-value |
| Model | 3604.326 | 9 | 400.4807 | 35.91889 | < 0.0001 |
| A-Temperature | 199.1158 | 1 | 199.1158 | 17.85858 | 0.0012 |
| B-Time | 26.92995 | 1 | 26.92995 | 2.415332 | 0.1461 |
| AB | 26.89641 | 1 | 26.89641 | 2.412324 | 0.1463 |
| A^2 | 90.60537 | 1 | 90.60537 | 8.126345 | 0.0146 |
| B^2 | 13.3291 | 1 | 13.3291 | 1.19548 | 0.2957 |
| A^2B | 33.87129 | 1 | 33.87129 | 3.037897 | 0.1069 |
| AB^2 | 11.97511 | 1 | 11.97511 | 1.074041 | 0.3205 |
| A^3 | 65.82808 | 1 | 65.82808 | 5.904084 | 0.0317 |
| B^3 | 0.203969 | 1 | 0.203969 | 0.018294 | 0.8947 |
| Residual | 133.795 | 12 | 11.14958 |  |  |
| Cor Total | 3738.121 | 21 |  |  |  |
